# Azoturia

**Published:** 1897-05

**Authors:** John R. Hart

**Affiliations:** Philadelphia


### AZOTURIA.

By John R. Hart, V.M.D.,
PHILADELPHIA.

[A synopsis of a paper read in 1883 by John R. Hart, V.M.D., of Philadelphia, with such modifications as circumstances and lapse of time would seem to warrant.

That paper contained the expression of an opinion, much criticised at that time, that azoturia was purely a liver and kidney disease, and the careful investigations of the subsequent fourteen years have but confirmed the opinion that azoturia is primarily due to a diseased liver.]

*Liver.* Physiology teaches us that the function of the liver is to secrete and to excrete. Any disease that retards these processes, or any derangement of this organ, lessens secretion of bile, which is one of the most important factors in the animal economy. The

[1] Read before the meeting of the Pennsylvania State Veterinary Medical Association, Philadelphia, March 2, 1897.

other function is to change the blood during its transit through the hepatic circulation, whereby it is fitted for its subsequent purposes in the animal. From autopsies I have made I am led to believe that more derangement to the system results from an inactive liver than we are aware of. Torpidity of this organ has a tendency at all times to cause indigestion. One of the chief functions of the liver is the secretion of bile for the purification of the blood by the ultimate excretion of effete matter, as you all know that bile is not discharged through an excretory duct or from a reservoir in the horse, but passes directly into the intestinal canal, where it mingles wth the chyme directly after it leaves the stomach. This shows the relation it has with the food with which it is mixed. We know it is alkaline in its reaction. Any alteration in its natural constituents has a deleterious effect on the general system.

Physiology teaches us that the liver arrests and renders harmless toxic substances which originate in the intestinal canal. If you have an inactive or diseased liver, this function is not performed and the toxins pass into the blood. The comparative toxicity of the portal and hepatic blood demonstrates that poisoning has no time to develop. The animal appears to succumb to an intestinal hyperæmia and consequent cerebral congestion in some cases. Professor Leonard Pearson, of the University of Pennsylvania, some six years ago called my attention to a gray gelding at that institution on which he had held an autopsy, which disclosed in the liver fatty degeneration, kidneys very much congested and large, lungs congested, and the entire alimentary canal in an intensely congested condition. The owner of this animal said that it had left the stable in apparently sound health three hours before its death.

I think we may therefore conclude that the liver is an organ of protection to the animal; that it arrests more or less toxic material in general, but not the whole, as a part passes out with the urine.

The liver is not the only organ which acts the part of a protector to the organism against poisons; we may consider as an auxiliary agent of protection rapidity of intestinal expulsion by the stools. Alterations in the intestinal contents result in the formation of hard fecal masses, which are harmless because absorption is no longer possible. We have a hurtful constipation, and from this the nervous system suffers—an example of reflex action.

Continued unusual pressure produces excessive activity of the nerves supplying the part; excitability is finally abolished, and paralysis of the nerve power occurs locally, as is seen in overdistention of the bladder. A constantly overloaded condition of the bowels may produce either of these local results on the nerve-filaments themselves. This effect travels backward to the motor ganglia in the lumbar cord, and defecation, being essentially a reflex act, when its directing centre is not sensitive to the controlling impulse of the brain, does not occur promptly and the constipation thus reacts upon the whole system. This produces a form of azoturia that is invariably fatal.

As for the biliary salts, they do not kill by direct intoxication alone; they dissolve and break up blood-globules and other cells, striated muscular fibres, and cells of the liver. They therefore cause anatomical lesions, and intoxication arises from the setting free of toxic substances which enter into the composition of the cellular elements. This intoxication develops but slowly. So long as there is functional activity of the kidneys, all is well; but if this activity ceases, then death may result from the other products of cellular destruction. It is said that if all the bile which the liver secretes passed directly into the blood, man would be poisoned by his own bile in eight hours. If all the urine that the kidneys secrete passed directly into the blood, man would be poisoned by his own urine in two days. We see the danger which results either from an impediment in the way of the elimination of bile or from its absorption. Fortunately more than one-half is eliminated in twenty-four hours by the digestive canal. What becomes then of the half of the bile which is not thrown out by the digestive canal? Does the liver destroy it? Does the tissue change it? These two hypotheses are possible, but are not demonstrated. What is demonstrated is that in the intestine a portion of the bile ceases to be absorbable. The coloring-matter and the biliary salts are metamorphosed, precipitated, or rendered insoluble; yet in certain morbid conditions bile may be absorbed in the liver itself at the margin of the hepatic cells. In these cases if the kidneys remain permeable they ward off intoxication; if they have ceased to be so, poisoning is the result.

The above facts lead to the conclusion that when from a faulty liver we can have, aside from liver and kidney, a third source of poison for the blood, as we do in azoturia (that is, putrefaction), not only that which arises from the imperfectly digested matter, but that which the presence of microörganisms in the intestinal tube incessantly maintains in the digestive canal, the conditions most favorable for the elaboration of poisons are realized. Therein are found nitrogenous substances already peptonized, and peptones are, as you know, excellent culture-media for microbes.

The conditions favorable for the maintenance of putrefaction are so numerous that we ask whether digestion can ever go on normally. Fortunately the stomach secretes, on the introduction of food, a juice—hydrochloric acid—which arrests fermentation.

Bile is regarded as capable of prolonging the arrest of fermentation, but it is capable of undergoing fermentation itself, or of putrefying. It can therefore only feebly oppose fermentation in the small intestine. At any rate, it can have no influence upon that which is actively carried on in the large intestine.

Thus we find the small intestine, on the one hand, and the large intestine particularly on the other, capable of passing products of putrefaction into the blood. But are the putrid substances toxic?

Gaspard in 1822 established the fact that putrid substances are toxic and that they are actually more so than substances arising from disassimilation. He injected into the veins of animals liquid from putrid blood of meat. He induced faintness, diarrhœa, hyperæmia of mucous membrane, then death. At the autopsy ecchymoses of the digestive canal were seen; also of cellular tissues and of muscles of heart, swelling of spleen and mesenteric glands, and congestion of lungs. All these conditions are found after death in azoturia.

We may have alterations in the liver, due to a congested condition, which, if continued or frequently repeated, would result in bilious contamination of the blood. Or perhaps from some previous disease we may have alterations in the liver that would have a tendency to prevent it from doing its proper work. Any inability of the liver or derangement of its functions would cause changes to take place in the alimentary canal, bile being in part reabsorbed into the blood, contamination following.

Now, what effect have the alterations mentioned, and what are the toxins? The organism contains poisons the origin of which we know—the destruction of cells, disassimilation of secretions, ingestion and putrefaction. The digestive canal contains poisons which come from ingesta, bile, and putrid material; its contents therefore should be toxic. Experimentation has demonstrated that they are toxic from the potash and ammonia, toxic from bile and putrid material. While you cannot demonstrate experimentally that the poison enters the blood, you can demonstrate that it leaves the blood. When bile ceases to flow into the intestines it passes into the blood. This problem cannot be solved. Any alteration in the liver caused by disease will prevent the excretion of effete material. Now if such is the case, where will this effete material go? Will nature allow it to so flow or be taken up by some channel whereby the blood will not become contaminated with it? It has been shown that it does not. The result is that the effete material collects in the blood. With what results? To act as powerful irritants in the animal circulation. This material first prevents the proper flow of the blood; second, causes a complete stasis; third, a dilatation of bloodvessels; fourth, a direct pressure upon the nerves; fifth, a complete loss of power from pressure on the nerves; sixth, decomposition.

In my paper on azoturia in 1883 I stated that azoturia was purely a liver and kidney disease. At the present date I feel more than convinced that azoturia is purely a liver and kidney disease. The question may be asked, Why should the kidney be a factor in this disease of the liver? In conjunction with the paralysis by dilatation of the renal arteries, caused by the effete material circulating in the blood.

Guitéras says any agent that causes an obstruction of the circulation, or nervous influence which may act by trophic derangement by causing circulatory disturbance, vasomotor action, the necrosed part may be absorbed, retained, or discharged. We have general excess of blood where there is a condition of plethora, a true hyperæmia; with this we have nervous influence. This may be due to paralysis of vaso-constrictors, whereby the outflow of blood is prevented.

Now, from a pathological standpoint there is really no disease of the blood, but rather of organs, and we study conditions of the blood and blood-making structures, mainly the bone-marrow, the lymph glands and spleen, the blood-destroying organs, of which the liver and spleen are important ones.

*Lymph glands.* Their functions are to form blood-corpuscles, to filter lymph—for this flows between the cells of the lymph-adenoid tissues. In this way injurious substances are prevented from entering the circulation at times.

Dr. Wood says hæmoglobin causes a dark cherry-brown color in blood. The enormous blood-pressure forces the capillary circulation through the tissues.

*Kidney.* Now, what part does the kidney take in this disease, as a preventive organ, and what part of the nerve-functions are involved, one with the other, to cause the disease of azoturia?

Pathologists usually divide the course of a disease into three stages or periods: First, that of increase; second, that of acme, in which the symptoms remain stationary; third, that of decline. But these stages are far from existing in all diseases or following one another in regular succession. In acute disease alone are they presented with distinctness and regularity. In azoturia which breaks out suddenly in its full force the first stage is wanting, and the record terminates abruptly in death without any period of decline.

It will be quite proper to attribute to it only one part of the phenomena which might supervene when the impermeability of the kidney is such that it can no longer eliminate the toxic substances produced by the organism in proportion to their formation. With a kidney acting well, things may not go further; but if the renal emunction is insufficient, we may see developed traces of uræmic intoxication through simple exaggeration of intestinal fermentation. While the kidney itself may not be really diseased, it will be sufficient, for the quantity of toxic material introduced into the blood should exceed the activity of the kidney and its ability to eliminate it.

The quantity of toxic matter eliminated by the kidney in twenty-four hours is, without doubt, in excess of what is necessary to kill the whole body.

Now, if the kidney is unable to do its work properly from some previous ill condition, or has been doing some extra work for the liver, the blood will quickly become contaminated, and the result will be azoturia, as if all the bile secreted in eight hours was introduced suddenly into the blood, we should see fatal nervous effects produced immediately; but as elimination is constantly being effected through the kidneys, and as the fibres of connective tissues are being incessantly colored, while the blood reabsorbs only gradually, nervous accidents are thus averted. The tissues serve to protect the organism against these poisons. Experiments have shown that this coloring-matter is ten times more poisonous than biliary matter. Salts become fixed.

When we consider the functions of the kidneys we are more able to understand the important work they are called upon to perform in azoturia. They eliminate all superfluous matter that may arise. Blood filled with effete material will stop the secretion.

It requires the kidney to be considerably diseased, for, owing to its permeability, it is sufficient alone to eliminate the poison formed by the organism in proportion to its production. Below this rate commences intoxication. But before this arises we see abnormal phenomena appear. First, albuminuria. Albuminuria is the accident of bad repute in diseases of the kidney.

The extreme cases belong chiefly to amyloid nephritis, because in such the liver, spleen, stomach, and intestines are diseased. All the organs whose function it is to transform the peptones of digestion into serum-albumin have undergone deterioration at the same time as the kidneys.

When such a large number of organs are diseased we can scarcely regard all the accidents which happen as due to renal impermeability. We cannot say that there is uræmia nor even kidney disease. There is a general disease of organs concerned in assimilation.

The inability of the kidney to do this extra amount of work is indirectly the cause of the paralysis, while the diseased liver, on the other hand, is the direct cause of the diseased kidney. The question may be asked, How is the liver the direct cause? From a pathological point, any derangement of the liver increases the labor of the kidneys.

The kidney becomes inactive from this source, and then refuses to perform its work. Functions are lost for the time being, with the result that the blood becomes contaminated with urea, as every aliment would become a poison if renal elimination was not the safeguard of the body.

(To be continued.)

